# Combining the use of Nuss procedure and rib fixation for severe flail chest: a case report

**DOI:** 10.1186/s12893-020-00747-2

**Published:** 2020-05-05

**Authors:** Quanwei Guo, Jinghui Zhang, Kaican Cai, Jianhua Zhang

**Affiliations:** 1 Department of Thoracic Surgery, Shenzhen Hospital, Southern Medical University, No.1333, Xinhu Road, Bao’an District, Shenzhen, 518101 Guangdong China; 2 Department of Thoracic Surgery, Nanfang Hospital, Southern Medical University, Guangzhou, 510515 Guangdong China; 3 Department of Thoracic Surgery, Wuwei People’s Hospital, Wuwei, 733000 Gansu China

**Keywords:** Severe flail chest, Nuss procedure, Rib fixation, Minimally invasive surgery

## Abstract

**Background:**

Severe flail chest is a life-threatening situation. The Nuss procedure is a new effective treatment for severe flail chest patients who cannot be weaned from prolonged mechanical ventilation in the last few years. However, the procedure is not suitable when there are multiple fractures in both the anterior and lateral chest walls. Here, we reported a rare case of severe flail chest in a patient who suffered multiple fractures in both the anterior and lateral chest walls in a traffic accident.

**Case presentation:**

A 49-year-old patient suffered severe flail chest by a steering wheel in a traffic accident with multiple fractures in both the anterior and lateral chest walls. In the beginning, the patient was administrated with mechanical ventilation because of acute respiratory distress syndrome (ARDS) for more than 1 week. Then the patient suffered from a severe lung infection and decreased blood oxygen saturation. After a multidiscipline discussion (MDT), three rib fixation plates were first used to rebuild the stability of lateral chest walls, then two Nuss bars were inserted to eliminate paradoxical movement in the anterior chest wall. Finally, the patient recovered smoothly after the combining procedure.

**Conclusions:**

Severe flail chest patients with both the anterior and lateral chest walls after trauma are in a life-threatening situation, and require an appropriate procedure to get out of danger in time. Rib fixation is an effective treatment when the fractured sites are few and the fractured area is small. The Nuss procedure is a new effective method for severe flail chest with multiple fractures in an anterior chest wall, which is also a minimally invasive and short time-consuming procedure. However, it does not suitable for the patient with multiple fractures in lateral chest walls. Combining the use of Nuss procedure and rib fixation can solve severe flail chest with multiple ribs and sternum fractures in both the anterior and lateral chest walls, and the outcome of this procedure is satisfying in the present rare case.

## Background

Flail chest, caused by multiple consecutive rib fractures with or without sternal fractures, is a life-threatening situation because of paradoxical movement, and its mortality estimates ranging from 9 to 20% [1, 2].

Rib fixation is an effective treatment for rib fractures. The Nuss procedure has been reported to repair pectus excavatum by elevating the depressed chest wall since 1998 [3]. It has been used for surgical treatment of severe flail chest recently, providing good outcomes whether flail chest is caused by trauma [4–9] or disease [10]. It is also a minimally invasive and short time-consuming procedure. However, the Nuss procedure is not suitable when there are multiple fractures in both the anterior and lateral chest walls, as unstable lateral chest walls can’t support and secure the Nuss bar. Here, we reported a rare case of severe flail chest in a patient who suffered multiple fractures in both the anterior and lateral chest walls in a traffic accident. Our report proved that combining the use of the Nuss procedure and rib fixation was a feasible and effective strategy for this situation with satisfying surgery outcomes.

## Case presentation

**Fig. 1:**
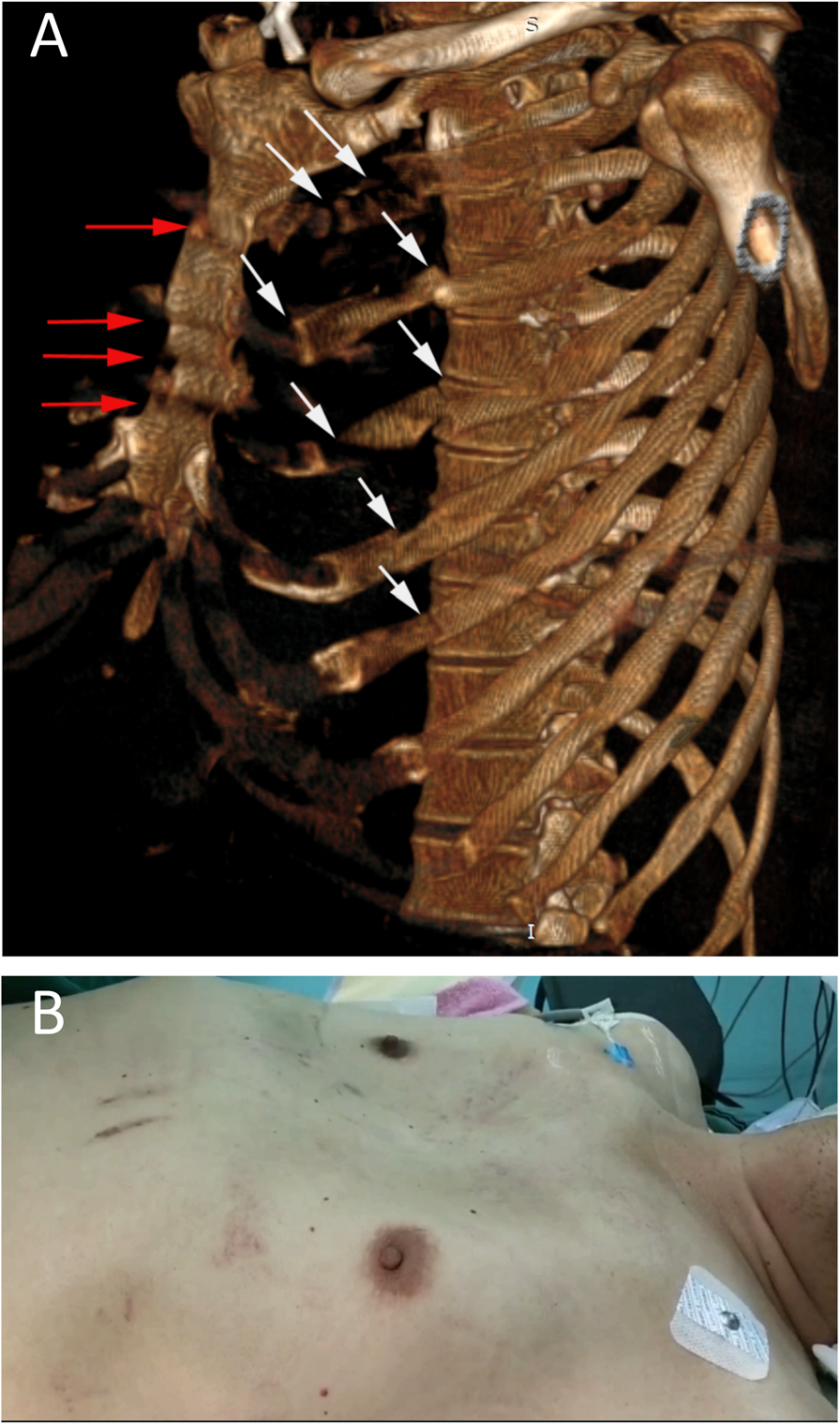
preoperative chest wall **a** preoperative bone three-dimensional reconstruction of the left chest wall: ribs fractures (white arrows) and sternal fractures (red arrows). **b** preoperative depressed anterior chest wall

A 49-year-old man was admitted to our emergency department after severe trauma to the chest wall by a steering wheel in a traffic accident. When he was sent to our hospital, his vital signs were as follows: respiratory rate 39 breaths/minute; heart rate 142 beats/minute; blood pressure 127/76 mmHg; blood gas pH 7.235; pCO_2_ 64.2 mmHg; and pO_2_ 56.5 mmHg. Chest computed tomography (CT) revealed multiple sternal fractures, bilateral multiple rib fractures from the first to the seventh ribs (Fig. 1a), and bilateral hemopneumothorax. The anterior chest wall was depressed due to multiple fractures (Fig. 1b). Closed thoracic drainage was performed bilaterally in the intensive care unit (ICU). The paradoxical movement gradually worsened, and respiratory failure emerged (Video S1).

The patient had been consistently administrated with ventilator-assisted ventilation for over 1 week, however, no improvement of paradoxical respiration was observed. More importantly, the patient suffered from a more severe lung infection and the blood oxygen saturation decreased obviously. Therefore, a multidisciplinary discussion including experts from departments of thoracic surgery, ICU, respiration, radiology, as well as anaesthesiology was organized. The physicians from the respiratory department and ICU insisted that the patient still required ventilator-assisted ventilation although it could not ameliorate severe abnormal breathing, extra antibiotic usage with effective lung care might be useful to suppress lung inflammation, the most important treatment was to perform chest wall fixation. With the three-dimensional bone reconstruction images of chest wall provided by the radiology department, the chief physician from the thoracic surgery department indicated that it was not suitable to perform conventional rib and sternum fixation in this case as the images clearly showed too many fracture sites in ribs (including costal cartilage), sternum, anterior as well as lateral chest wall (Fig. 1a, Fig. S1), thus this method could not establish a stable chest wall, and time-consuming, traumatic, more bleeding and costly. We noticed the use of Nuss procedure in trauma had been previously reported [4–9]. It was a new effective treatment for severe flail chest patients who couldn’t survive without prolonged mechanical ventilation. The most important advantages for the Nuss procedure were minimally invasive and much less time-consuming. However, it was not suitable when there were combined fractures in both the anterior and lateral chest walls as the Nuss bar required a stable lateral chest wall to guarantee the physical support, which the patient lacked. Therefore, neither the Nuss procedure nor rib fixation could completely fix the chest wall and eliminate abnormal breathing. We then proposed a strategy to combine the Nuss procedure with rib fixation. First, the rib fixation rebuilt a stable lateral chest wall, then the Nuss procedure stabilized the front chest wall. This strategy was also supported by the physicians from the anaesthesiology department as he mentioned that the conventional rib and sternum fixation could severely influence the patient’s respiratory and circulatory system during the operation. Finally, combining the use of the Nuss procedure and rib fixation was determined after the multidisciplinary discussion.

**Fig. 2:**
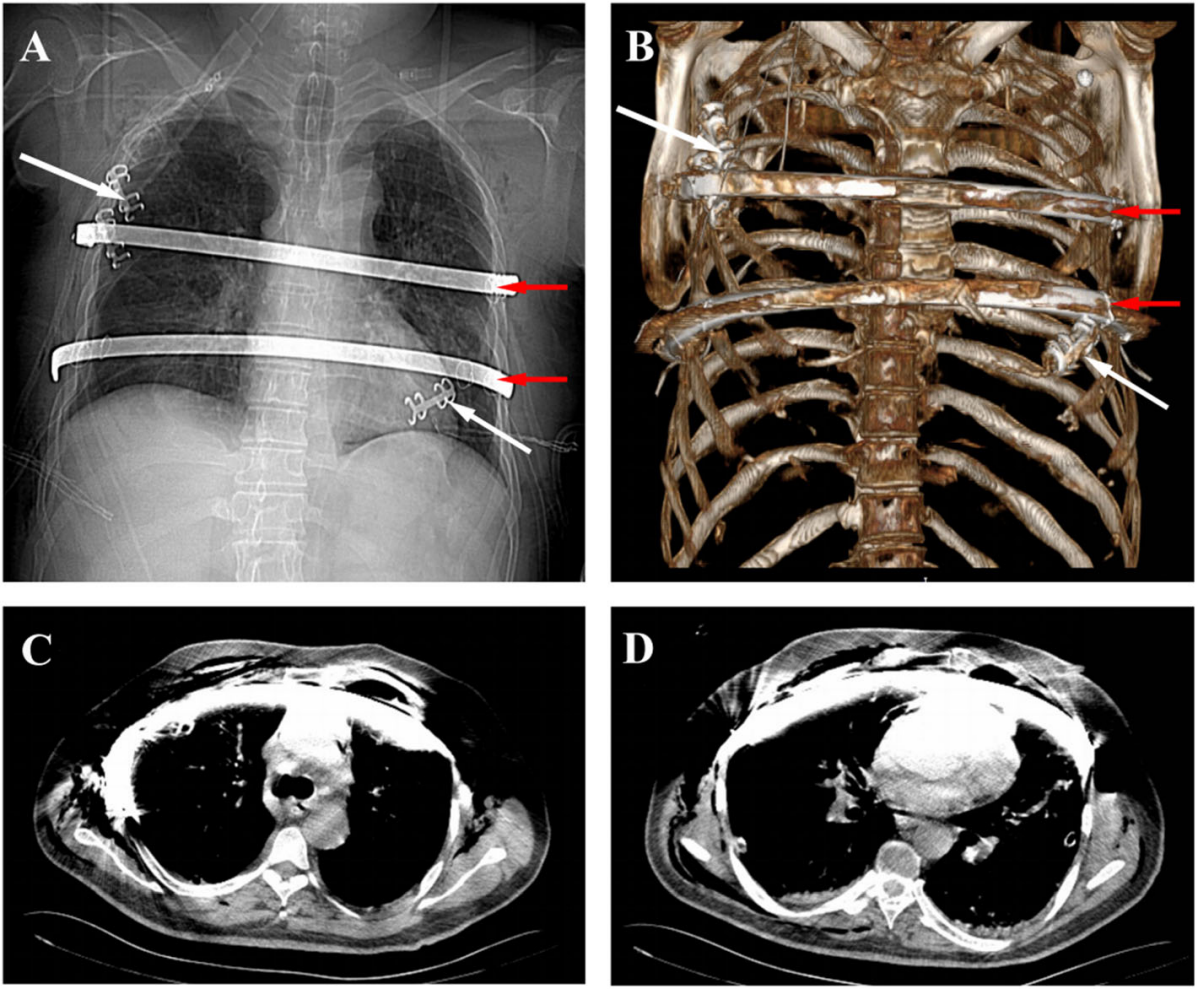
postoperative chest wall **a** postoperative chest X-ray with rib fixation plates (white arrows) and Nuss bars (red arrows) in place. **b** postoperative bone three-dimensional reconstruction of the chest wall with rib fixation plates (white arrows) and Nuss bars (red arrows) in place. **c** upper bar in the third intercostal space **d** lower bar infigure the fifth intercostal space

First, the right third and fourth and the left fifth fractured lateral ribs were stabilized using rib fixation plates (Seemine SMA Co., LTD, Gansu, China) to stabilize both lateral chest walls (Fig. 2a, b, white arrows). Then the thoracoscopy-assisted Nuss procedure was performed. Two Nuss bars (GRINM Advanced Materials Co., LTD, Beijing, China) were inserted into the third and fifth intercostal spaces of both sides for elevating and stabilizing the depressed mid sternum and fractured ribs at the anterior chest wall respectively to avoid the fractured sites of the ribs (Fig. 2a, b, red arrows). The Nuss procedure process was as follows: one 40 cm Nuss bar was bent into a symmetric arc shape. Two skin incisions (1.5 cm) were made on both lateral chest walls in the mid-axillary line at the third intercostal space. Submuscular tunnels were made the outside pleural entry and exit points. The right entry point was punctured with an introducer, and a 1 cm thoracoscope was placed into the pleural cavity. The mediastinum was dissected under direct vision. The exit point at the left side was punctured under direct visual guidance as well, and a 32F chest tube passed through the tunnel created by the introducer. The bar was positioned by following the guidance of the chest tube. The Nuss bar was rotated, and the depressed anterior upper chest wall was elevated. However, the shape of the anterior lower chest wall still had depression resulting from the large fractured areas, and paradoxical movement existed too. Therefore, another 40 cm Nuss bar was inserted at the fifth intercostal space with the same method. Two bars finally were fixed on the stable lateral ribs with steel wires. Finally, two Nuss bars and three rib fixation plates simultaneously exert sustained support, and the shape of the chest wall was nearly perfect (Fig. 2c, d).

No complications occurred during the procedure (surgical time 85 min, bleeding volume 50 mL). Paradoxical respiration was eliminated immediately postoperation. The patient was weaned from mechanical ventilation on the third postoperative day. He recovered smoothly and was discharged 2 weeks after the operation. No short-term complications were found except pain and activity limitations, and the pain was blocked using epidural anaesthesia after the operation. Three months later, the patient had no pain and activity limitations, but only complained numbness at the surgical incisions. One year later, the patient lived a normal life without any adverse events. We have scheduled a completed examination including chest CT scan and three-dimensional bone reconstruction for this patient, the Nuss bars and rib fixation plates will be removed immediately once the chest wall fully recovered.

## Discussion and conclusions

Flail chest, caused by the fractured ribs and sternum, is in an unstable state, which can lead to lethal respiratory failure because of paradoxical movement. Respiratory failure often requires positive mechanical ventilation until the stability of the chest wall is restored. However, it can cause many other complications. It has been verified that surgical stabilization can reduce the incidence of ventilator-induced complications, decrease the duration of mechanical ventilation, shorten hospital length of stay and reduce the cost for flail chest patients [11].

Using rib fixation plates firstly comes into our minds when we encounter a patient with rib fractures [12]. However, it is not suitable for those severe patients who have too many fractured sites and large fractured areas both in ribs and sternum, especially with cartilage fractures. The patient described in this study was not suitable for normal rib fixation surgery.

The Nuss procedure has been performed to repair pectus excavatum by elevating the depressed chest wall since 1998 [3]. The depression of the flail chest is similar to the shape of pectus excavatum. Therefore, the Nuss procedure has been used for surgical treatment of severe flail chest, whether it is caused by trauma [4–9] or other diseases [10]. The most advantage of the Nuss procedure for flail chest is minimal invasion, short time-consuming and good outcomes. However, it is only suitable for patients with lateral chest wall stability, because both the fixation and force points of the Nuss bars are located on the lateral chest wall. An unstable lateral chest wall cannot support and secure the Nuss bar. In the case presented here, the patient suffered severe flail chest due to a traffic accident with multiple ribs and sternal fractures in both anterior and lateral chest walls, and they were all in an unstable state. Since the large depression area and the multiple fractured sites on the ribs and mid sternum, neither rib fixation nor the Nuss procedure alone could rebuild the floating chest wall. Therefore, we decided to combine the use of Nuss procedure and rib fixation for this severe flail chest patient after an MDT. Firstly, rib fixation plates were used to fix fractured ribs in the lateral chest wall, which could provide a steady lateral chest wall, then inserted a Nuss bar at the third intercostal space to assist elevation of the depressed anterior chest wall as well as the depression in pectus excavatum. However, the shape of the anterior lower chest wall still had depression due to large fractured areas, as well as the paradoxical movement. Therefore, we inserted another Nuss bar at the fifth intercostal space with the same approach. Finally, two Nuss bars and three rib fixation plates simultaneously exert sustained support, and the shape of the chest wall was nearly perfect. The patient recovered smoothly without complications after the operation, and the outcome of the combining procedure was satisfying.

Although combining the Nuss procedure and rib fixation has its contraindications and does not meet the requirements for anatomical relocation, it helped the flail chest patient to survive without mechanical ventilation or any severe adverse events.

In summary, this report aims to advocate that combining the use of Nuss procedure and rib fixation would be a feasible strategy when we treat such severe flail chest patients with multiple ribs and sternum fractures in both the anterior and lateral chest walls.

## Supplementary information

**Additional file 1: Figure S1.** preoperative bone three-dimensional reconstruction of the left chest wall.

**Additional file 2: Video S1**. palpable paradoxical respiration.

## Data Availability

The datasets used and/or analysed during the current study are available from the corresponding author on reasonable request.

## Abbreviations

ARDS: Acute respiratory distress syndrome
CT: Computed tomography
ICU: Intensive care unit
MDT: Multidiscipline discussion
