# Supplementary figures and images for: Combining the use of Nuss procedure and rib fixation for severe flail chest: a case report
Source: BMC Surg. 2020 May 5;20:87. doi: 10.1186/s12893-020-00747-2 (PMC7201783; doi:10.1186/s12893-020-00747-2)

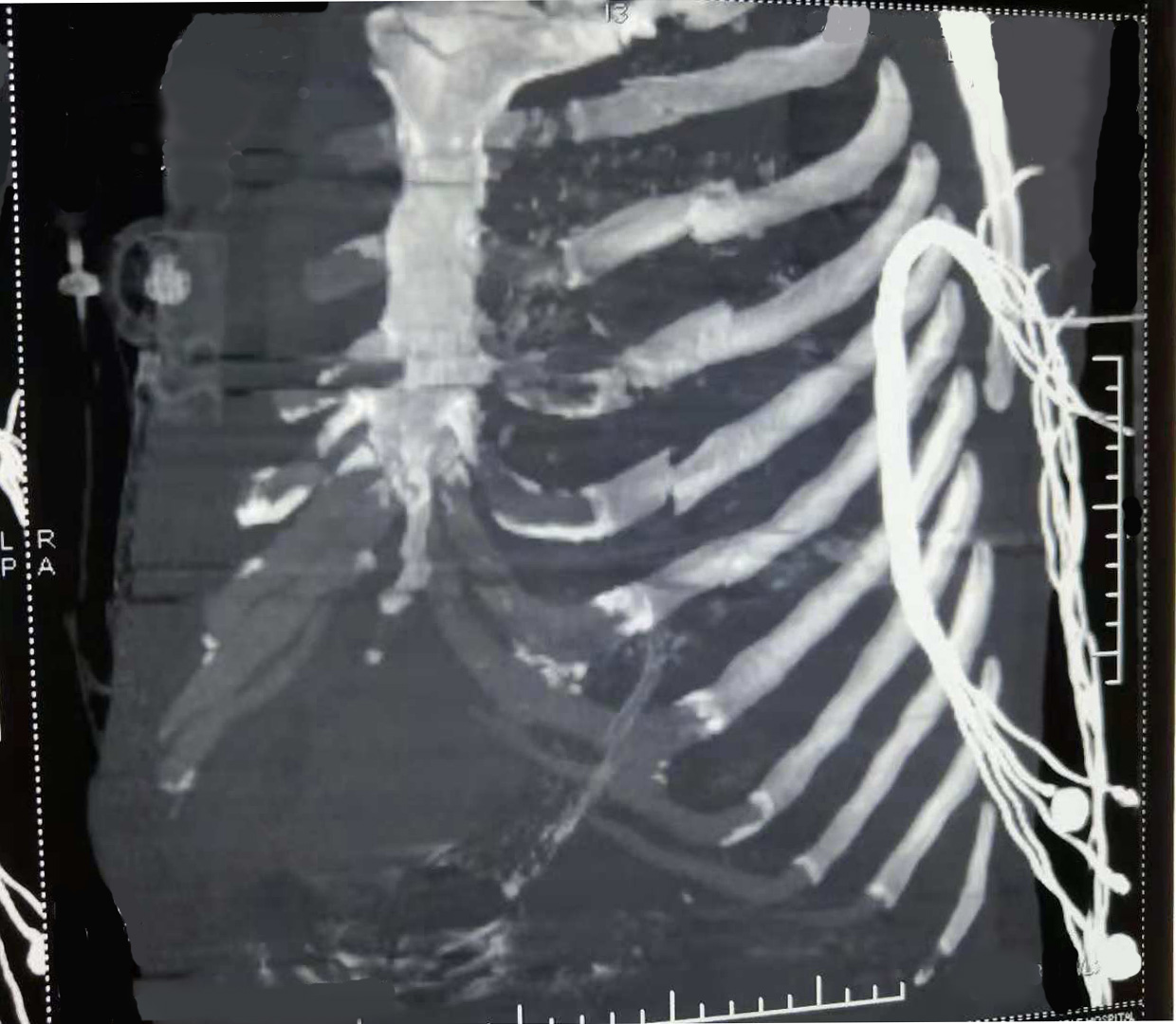

Supplement: Supplementary file 1 — Additional file 1: Figure S1. preoperative bone three-dimensional reconstruction of the left chest wall. [file 12893_2020_747_MOESM1_ESM.tif]
